# Biomolecular Evaluation of Piceatannol’s Effects in Counteracting the Senescence of Mesenchymal Stromal Cells: A New Candidate for Senotherapeutics?

**DOI:** 10.3390/ijms222111619

**Published:** 2021-10-27

**Authors:** Nicola Alessio, Tiziana Squillaro, Ida Lettiero, Giovanni Galano, Roberto De Rosa, Gianfranco Peluso, Umberto Galderisi, Giovanni Di Bernardo

**Affiliations:** 1 Department of Experimental Medicine, Biotechnology and Molecular Biology Section, University of Campania “Luigi Vanvitelli”, 80138 Naples, Italy; nicola.alessio@unicampania.it (N.A.); squillarot@yahoo.it (T.S.); idalettiero95@gmail.com (I.L.); 2 ASL Napoli 1 Centro P.S.I. Napoli Est-Barra, 80147 Naples, Italy; galanog@inwind.it (G.G.); robertoderosa@libero.it (R.D.R.); 3 Research Institute on Terrestrial Ecosystems, CNR, 80131 Naples, Italy; gianfranco.peluso@cnr.it; 4 Center for Biotechnology, Sbarro Institute for Cancer Research and Molecular Medicine, Temple University, Philadelphia, PA 19122, USA

**Keywords:** mesenchymal stem cells, polyphenols, senescence, senolytics

## Abstract

Several investigations on senescence and its causative role in aging have underscored the importance of developing senotherapeutics, a field focused on killing senescent cells and/or preventing their accumulation within tissues. Using polyphenols in counteracting senescence may facilitate the development of senotherapeutics given their presence in the human diet, their confirmed tolerability and absence of severe side effects, and their role in preventing senescence and inducing the death of senescent cells. Against that background, we evaluated the effect of piceatannol, a natural polyphenol, on the senescence of mesenchymal stromal cells (MSCs), which play a key role in the body’s homeostasis. Among our results, piceatannol reduced the number of senescent cells both after genotoxic stress that induced acute senescence and in senescent replicative cultures. Such senotherapeutics activity, moreover, promoted the recovery of cell proliferation and the stemness properties of MSCs. Altogether, our findings demonstrate piceatannol’s effectiveness in counteracting senescence by targeting its associated pathways and detecting and affecting P53-dependent and P53-independent senescence. Our study thus suggests that, given piceatannol’s various mechanisms to accomplish its pleiotropic activities, it may be able to counteract any senescent phenotypes.

## 1. Introduction

As a process that causes detrimental physical and physiological changes over time, aging is often associated with several chronic diseases that account for the lion’s share of overall morbidity and mortality. During aging, the primary processes at work are low-grade inflammation, the dysfunction of macromolecules and cellular organelles, the impairment of stem cell functions, and senescence [1].

Among those processes, senescence induces permanent cell cycle arrest, accompanied by the loss of cellular functions, which is caused by genotoxic stresses occurring over the course of life. Extensive evidence correlates senescence to aging, given its contribution to reduced tissue functions, cell renewal, and homeostasis. Indeed, senescent cells accumulate in the adipose tissue of people suffering from diabetes and/or age-related metabolic diseases and in the atherosclerotic vessels, osteoarthritic joints, and lungs of people with idiopathic pulmonary fibrosis or other age-associated chronic diseases [1,2]. Senescence also exerts conflicting effects on cancer. On the one hand, it may be part of an anticancer mechanism that induces the arrest of damaged or otherwise transformed cells; on the other hand, because the process occurs during aging, senescent cells may promote and/or sustain the growth of cancer [1,2]. Indeed, cancer cells can coax senescent cells to secrete factors for their survival, thus abrogating the senescence anti-cancer effect [3,4].

Senescent cells lose their primary functions and acquire new ones, they primarily accomplishing the latter by secreting hundreds of molecules in what is collectively known as the senescence-associated secretory phenotype (SASP) [5]. Several growth factors, proinflammatory cytokines, chemokines, and many other bioactive factors contribute to SASP, which acts as a so-called “threat signal”, stimulates normal cells to senesce, and reinforces senescence via autocrine signaling [5].

In recent years, new therapeutic strategies have been devised to fight aging and its related chronic diseases. Such approaches aim to reduce the accumulation of senescent cells within tissues and the negative activities of SASP (e.g., stimulation of inflammation, nonfunctional remodeling of the extracellular matrix, and promotion of senescence). A pioneering investigation demonstrating that the strategy may be effective involved studies on a transgenic mouse model, in which the specific elimination of putative senescent cells accumulated within tissues delayed and/or attenuated some age-related diseases [6].

A new class of drugs dubbed senotherapeutics is now under scrutiny for delaying aging and associated diseases as part of a strategy aimed at promoting healthy aging and well-being during the last phase of life. Senotherapeutics include senomorphics and senolytics, the latter of which entails selectively killing senescent cells by exploiting their high resistance to apoptosis. Meanwhile, senomorphics may prevent the onset of senescence following genotoxic stress either by interfering with signals inducing senescence or by blocking SASP. Other types of senomorphics, by contrast, may cause the reversion of the phenotype of senescent cells [7,8].

As natural compounds widely distributed in edible plants and fruits, polyphenols can be divided into four subclasses such as phenolic acids, flavonoids, stilbenes, and lignans. All compounds in those subclasses may have health-promoting effects when consumed daily as part of one’s diet. Mounting evidence shows that drugs based on those compounds may be of interest for neurological, cardiological, and metabolic diseases, as well as for treating cancer and aging-related pathologies [9,10,11]. Studies investigating the beneficial effects of resveratrol, a stilbene compound, and its derivatives on human health have demonstrated that resveratrol may protect cells from the onset of senescence primarily via its antioxidant properties [12,13]. Nevertheless, because polyphenols such as resveratrol have complex molecular mechanisms of action on human health, their pleiotropic activities depend on several parameters, including dosage. It has also been shown that polyphenols may act either as senomorphic or senolytic agents [8,9].

Although otherwise similar to resveratrol, piceatannol (3,5,4′,3′-tetrahydroxystilbene) contains a single hydroxyl group at the 3′-carbon (3,5,4′-trihydroxystilbene) that affords the compound more powerful antioxidant activity, a longer half-life, and greater bioavailability than resveratrol [9,14]. Knowing that, we evaluated the effects of piceatannol on the acute and chronic (i.e., replicative) senescence of human mesenchymal stromal cells (MSCs), a heterogeneous population of stem cells, progenitor cells, fibroblasts, and stromal cells. MSCs secrete several factors that regulate immune functions and the renewal processes of various kinds of tissue. As the senescence of MSCs may have profound negative consequences for human health, evaluating senotherapeutics that counteract that process is greatly needed [15].

## 2. Results

### 2.1. Piceatannol at Micromolar Concentrations Reduced the Senescence of MSCs

In the great majority of investigations, the micromolar scale is the maximum order of magnitude for in vitro treatment with stilbenes, both because higher doses have unspecific in vitro cytotoxicity and given in vivo data indicating safe oral administration at that magnitude without severe side effects [9,14,16].

In our investigation, we first treated MSCs at 10 DIV (days in vitro) with increasing doses of piceatannol and evaluated drug doses below the inhibitory concentration able to kill 50% of cells (IC_50_) (Table 1). Ultimately, we chose 10 μM piceatannol as the maximum in vitro dose that did not produce nonspecific cell death. Our results are in concordance with previous in vitro data [17,18].

**Figure 1:**
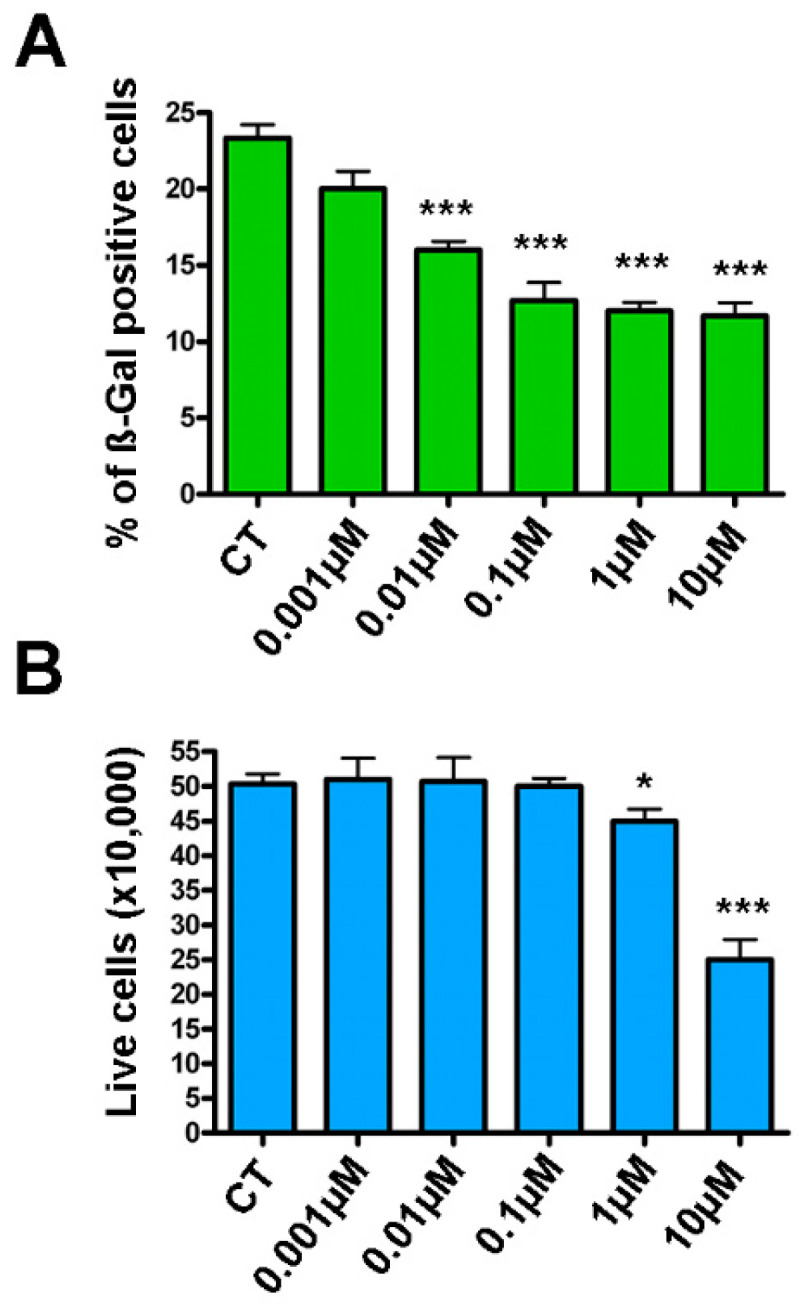
Effects of piceatannol on acute senescence. MSCs at 10 DIV were treated for 30 min with 300 μM hydrogen peroxide to induce acute senescence, which was evaluated by beta-gal assay 48 h later. Next, senescent cultures were incubated with different amounts of piceatannol for another 48 h. The upper chart (**A**) shows the percentage of senescent cells at the end of piceatannol treatment. The lower chart (**B**) reports the cell viability, as detected via CCK-8 assay, of cultures treated with piceatannol. Data are expressed in arbitrary units and shown with standard deviation (SD), n *=* 3. *** *p* < 0.001 and * *p* < 0.05 indicate statistical significance between the control and treated samples. CT = control samples.

Next, we treated MSCs with hydrogen peroxide in order to induce acute senescence. Incubation with the genotoxic molecule induced senescence in 10–20% of cells as detected by beta-galactosidase (beta-gal) assay. With those results in hand, we incubated the senescent cultures with increasingly greater doses of piceatannol for 48 h, after which we reassessed the percentage of senescent cells. Piceatannol reduced the number of senescent cells in culture at all of the tested concentrations except the lowest (i.e., 0.001 μM), as shown in Figure 1A. Piceatannol appeared to act as a senomorphic agent in the range of 0.01–1 μM, while at 10 μM it seemed to have senolytic activity. In fact, at low concentrations, piceatannol reduced senescence without affecting cell death, whereas at 10 μM we detected a significant reduction in the number of cells (Figure 1B). 

### 2.2. Piceatannol Effectively Act Both on Acute and Chronic Senescence

**Figure 2:**
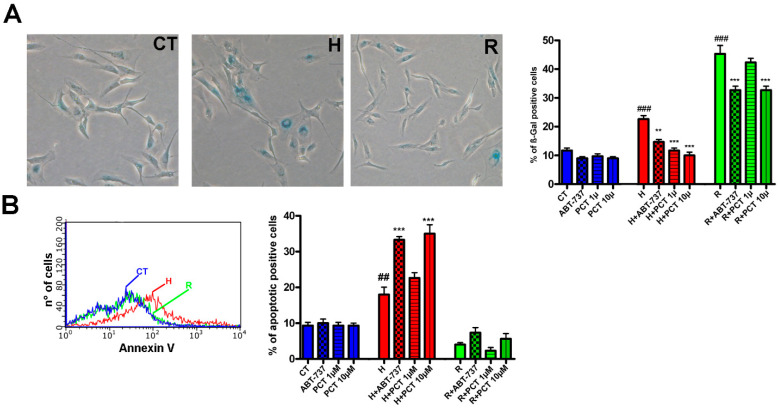
Senolytic versus senotherapeutic effects of piceatannol. Panel (**A**): MSCs at 10 DIV were treated for 30 min with 300 μM hydrogen peroxide to induce acute senescence, which was evaluated by beta-gal assay 96 h later. Senescence was also determined in 30 DIV cultures. The pictures show representative images of beta-gal staining in control cultures at 10 DIV (CT), in 10 DIV samples treated with hydrogen peroxide (H), and in replicative senescent cultures at 30 DIV (R). The chart on the right shows the percentage of senescent cells in healthy cells (CT) treated with ABT-737 or PCT (blue bars), in peroxide hydrogen-treated cultures (red bars) and in replicative senescent cultures (green bars). Panel (**B**): Apoptosis levels were detected in control cultures at 10 DIV (CT), in 10 DIV samples treated with hydrogen peroxide (H), and in replicative senescent cultures at 30 DIV (R). The picture shows the flow cytometry chart of annexin V assay to detect apoptosis. The chart on the right shows the percentage of apoptotic cells in healthy cultures (CT) treated with ABT-737 or PCT (blue bars), in peroxide hydrogen-treated cultures (red bars) and in replicative senescent cultures (green bars). Data are shown with standard deviation (SD) n = 3. The ^###^ *p* < 0.001 and ^##^ *p* < 0.01 are the statistical significances between control (CT) and peroxide hydrogen treated samples (H) or replicative senescent cultures (R). The *** *p* < 0.001 and ** *p* < 0.01 are statistical significances between control and treated samples.

To further investigate piceatannol’s effect on senescence, we tested doses of 1 μM and 10 μM as a means to discriminate between potential senolytic and senomorphic activity. As senolytics induces apoptosis and, in turn, eliminates senescent cells, we determined the level of apoptosis in senescent cultures treated with piceatannol. As a reference for senolytics activity, we used ABT-737, which kills senescent cells by inhibiting certain anti-apoptotic pathways [19]. We broadened the investigation by assessing piceatannol’s effect on the chronic (i.e., replicative) senescence of MSC cultures at 30 DIV. In a quantity of 1 μM, ABT-737 reduced senescence both in samples treated with hydrogen peroxide and in replicative senescent cultures (Figure 2A). Meanwhile, 10 μM piceatannol also reduced senescence in replicative cultures but was ineffective at lower concentrations (Figure 2A). Those results suggest that the reduction in senescence is associated with an increase in apoptosis (Figure 2B). Notably, the piceatannol did not induce significant changes in apoptosis and senescence levels in healthy cultures (Figure 2A,B).

**Figure 3:**
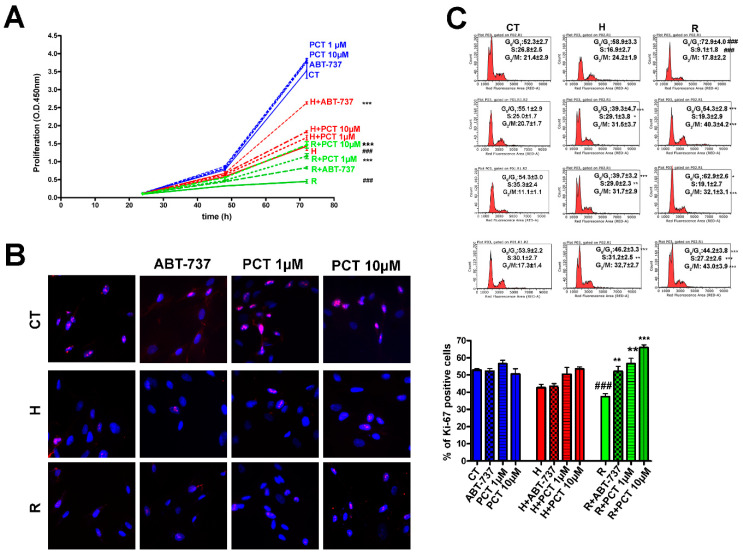
Cell cycle profile and proliferation of cells treated with piceatannol. MSCs at 10 DIV were treated for 30 min with 300 μM hydrogen peroxide to induce acute senescence. After 48 h, senescent cultures were incubated with ABT-737 or piceatannol for another 72 h. Alternatively, MSCs at 30 DIV (i.e., replicative senescent cultures) were incubated with ABT-737 or piceatannol for 72 h. Panel (**A**) shows the proliferation of healthy cultures (blue lines), acute (red lines) and chronic (green lines) senescent cultures with or without ABT-737 or piceatannol. Data are expressed as 450 nm O.D. The percentage of cycling cells (i.e., Ki67 positive) and the cell cycle flow cytometry charts of cultures treated as described above are reported in Panels (**B**,**C**), respectively. In Panel (**B**), the pictures show representative images of Ki67 staining (i.e., in red) in control cultures at 10 DIV (CT), in 10 DIV samples treated with hydrogen peroxide (H), and in replicative senescent cultures at 30 DIV (R). The chart on the right shows the percentage of Ki67(+) cells in healthy cultures (CT) treated with ABT-737 or PCT (blue bars), in peroxide hydrogen treated cultures (red bars) and in replicative senescent cultures (green bars). Statistical data are shown with standard deviation (SD) n = 3. In panel (**B**,**C**) the *** *p* < 0.001, ** *p* < 0.01 and * *p* < 0.05 levels are statistical significances between control and samples with piceatannol or ABT-737. In panel (**B**,**C**), the ^###^ *p* < 0.001 are statistical significances between control (CT) and peroxide hydrogen-treated samples (H) or replicative senescent cultures (REP). In the panels, the statistically significant values refer to 72 h treatments.

Eliminating senescent cells should improve the healthiness of MSC cultures, which can be evaluated by determining the cell cycle profile and the proliferation of cells in the presence of piceatannol. The proliferation in cultures treated with hydrogen peroxide significantly increased in the presence of ABT-737 or piceatannol, both at concentrations of 1 μM and 10 μM (Figure 3A). In the replicative senescent culture, however, piceatannol was more effective than ABT-737 in reviving cell proliferation (Figure 3A). Of note, the piceatannol did not induce significant changes in proliferation in healthy cultures (Figure 3A).

Those results concur with the outcomes of a Ki67 staining assay conducted to detect the percentage of cycling cells (i.e., Ki67-positive cells) compared with resting ones (i.e., Ki67-negative G_0_ cells). Indeed, cultures treated with ABT-737 showed a slight albeit non-significant increase in the percentage of cycling cells, whereas piceatannol-treated samples showed significant increases in cycling cells (Figure 3B). The piceatannol did not induce significant changes in the Ki67 level in healthy cultures (Figure 3B).

The analysis of cell cycle profiles further clarified piceatannol’s positive effect on senescent MSC cultures (Figure 3C). Cells in the S phase in peroxide-treated cultures amounted to 16.9% compared with 26.8% in the untreated samples. Incubation with 1 μM or 10 μM piceatannol increased those percentages to 29.0% and 31.2%, respectively. Results in the replicative senescence samples were even more remarkable. In the replicative senescent cultures, 9.1% of cells were in the S phase, and that percentage rose to 19.1% and 27.2% following treatment with 1 μM and 10 μM piceatannol, respectively (Figure 3C). The piceatannol did not induce significant changes in the cell profile pattern in healthy cultures (Figure 3C).

**Figure 4:**
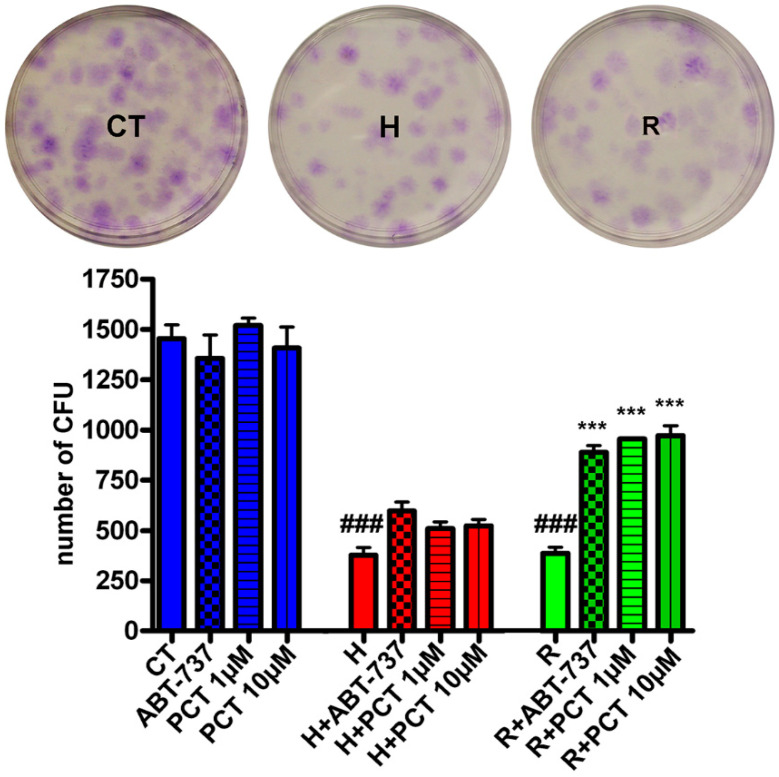
Stemness of MSCs treated with piceatannol. MSCs at 10 DIV were treated for 30 min with 300 μM hydrogen peroxide to induce acute senescence. After 48 h, senescent cultures were incubated with ABT-737 or piceatannol for another 72 h. Alternatively, MSCs at 30 DIV were incubated with ABT-737 or piceatannol for 72 h. Following treatment with drugs, the cultures were incubated at low density in order to determine the CFU potential. Representative pictures of CFU assays performed on the control and senescent MSC cultures are shown. The histogram shows the CFU number in healthy cultures (CT) treated with ABT-737 or PCT (blue bars), in peroxide hydrogen-treated cultures (red bars) and in replicative senescent cultures (green bars). The ^###^ *p* < 0.001 indicates statistical significance between the control and senescent samples. The *** *p* < 0.001 indicates statistical significance between the control samples and samples treated with piceatannol or ABT-737.

Senescence affects the stemness properties of stem cell populations present in MSC cultures. As senotherapeutics should majorly impact the self-renewal and multipotential differentiation properties of stem cells in order to be effective, we evaluated whether piceatannol promoted the recovery of MSCs’ self-renewal by performing a CFU assay (Figure 4). As a result, we observed that piceatannol and ABT-737 both increased the number of CFU clones in cultures treated with hydrogen peroxide or in the replicative senescent cultures (Figure 4). The piceatannol did not induce significant changes in CFU clone number in healthy cultures (Figure 4).

### 2.3. Piceatannol Affect Molecular Pathways Associated with Acute and Chronic Senescence

Senescent cells are heterogeneous, for not all of them express the same genes or present the same phenotype. Moreover, the signaling pathways associated with the onset and maintenance of senescence depend on several parameters, including genotoxic stimulus, cell type, and species. In past research, we identified signaling pathways associated with the acute and chronic senescence of human MSCs, pathways that can be roughly divided into P53- and/or RB1- or RB2-dependent signaling [20,21].

**Figure 5:**
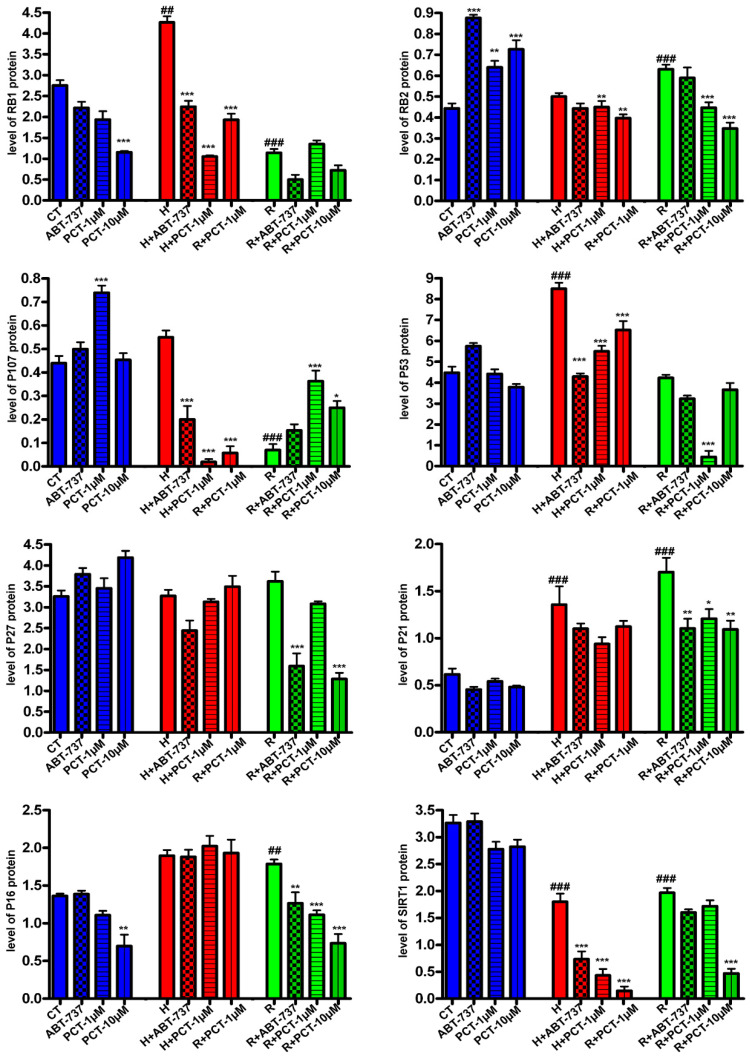
Signaling pathways associated with senescence and the effect of piceatannol treatment. The charts show the expression of RB1/P105, RB2/P130, P107, P53, P27/KIP1, P21/CIP1, P16/INK4A and SIRT1 in control samples (CT) and in samples treated with hydrogen peroxide (H) or in replicative senescence cultures (R). The charts show the densitometric analysis of Western blot bands shown in Supplementary file S3. The expression of proteins of interest was normalized on GAPDH, chosen as the control. The ^###^ *p* < 0.001 and ^##^ *p* < 0.01 levels indicate statistical significance between the control and senescent samples. The *** *p*<0.01 ** *p* < 0.01 and * *p* < 0.05 indicate statistical significance between the control samples and samples treated with piceatannol or ABT-737.

In our study, we evaluated how and to what extent piceatannol may interfere with those signaling pathways. In MSC cultures treated with hydrogen peroxide, we detected increased P53, RB1, P21 and P16 expression, whereas we observed the upregulation of RB2, P21, P27, and P16 in the replicative senescent samples. In these samples we also detected a decrease in P53 level (Figure 5 and Supplementary File S3). Treatment with piceatannol significantly decreased the expression of RB1, and P21 in samples treated with hydrogen peroxide. The 10 μM piceatannol also decreased the P53 level. Moreover, piceatannol treatment was able to decrease the levels of RB2, P27, and P16 in chronic senescent cultures (Figure 5 and Supplementary File S3). However, in the latter samples, piceatannol increased the expression of P107, a protein in the retinoblastoma family associated with cycling cells [22]. 

### 2.4. How Does Piceatannol Interfere with Senescence Signaling Pathways?

The effect of resveratrol in counteracting senescence is mainly associated with regulation of SIRT1 expression and activity [23,24]. SIRT1 is an NAD-dependent deacetylase, which has multiple roles in cell biology. This enzyme can promote cell survival by blocking apoptosis and can preserve cells from senescence phenomena [25]. The anti-apoptosis and anti-senescence role played by SIRT1 is based mainly on negative modulation of P53-related pathways through deacetylation and inactivation of P53 [25,26,27].

We evaluated if the senotherapeutics activity of piceatannol may act through SIRT1. In healthy MSCs, the piceatannol did not modify the protein level of SIRT1 while, in senescent cultures, which showed a reduced SIRT1 expression compared to control cultures, the piceatannol further decreased the SIRT1 level (Figure 5 and Supplementary File S3). Of note, ABT-737 also reduced the SIRT1 expression. 

## 3. Discussion

### 3.1. Healthy Aging and Senotherapeutics

Healthy aging is the process of improving and preserving health, social, mental wellness, independence, and quality of life among older adults [28]. As biomedical and other scientific advances continue to facilitate the extension of human lifespans, they exponentially increase the population of people aged 65 years or older, a key challenge for whom is finding ways to sustain, if not improve, the quality and length of life. To that end, medical and social policies to promote healthy aging are in high demand. 

Several biomedical findings have illuminated the primary biological phenomena that cause the onset of aging and the related pathologies. In that context, many studies have addressed senescence, for senescent cells have a causative role in aging and age-associated diseases. Therefore, the development of senotherapeutics that specifically kills senescent cells and/or prevents their accumulation within tissues and organs could have significant therapeutic effects, including decelerated aging and improved resilience. Although several classes of molecules, whether synthetic or natural compounds, have been scrutinized as potential senotherapeutics [1,8], the development of senotherapeutics remains in its infancy and faces many obstacles before effective drugs can be created. Those hurdles stem from the intrinsic nature of senescence, for senescent cells can vary greatly in terms of markers, physiology, and the secretion of SASP factors, as well as exploiting different pathways to maintain their status and resist apoptosis signals [7,8,21]. In those contexts, drugs aimed at reducing or blocking senescence in stem compartments are paramount, because the impairment of stem cell functions can lower the renewal capacity of tissues and, as a consequence, reduce their very functionality.

### 3.2. Piceatannol Counteracts Acute and Chronic Senescence

We evaluated the effect of piceatannol, a natural polyphenol, on the senescence of MSCs, which play a key role in the body’s homeostasis. We chose a molecule in the polyphenol family for numerous reasons. For one, those compounds are prevalent in human diets, either in food or as supplements, and several studies have confirmed their tolerability and absence of severe side effects, even at high doses [9,10,11]. There are several studies showing that, at concentrations above 25–50 μM, the piceatannol may induce in vitro cell death in different cell types, while, at concentrations below 10 μM, this compound may have cytoprotective effects [17,29,30]. Our data are in concordance with these findings, since we detected the cytotoxic effect on healthy MSCs at 100 μM and not at the lower concentrations we evaluated.

Another interesting aspect of polyphenols is that they may either prevent senescence or induce the death of senescent cells. Last, they have different mechanisms of action to accomplish their pleiotropic activities, some of which may be able to counteract the different senescent phenotypes.

We have demonstrated that piceatannol can reduce the number of senescent cells both after genotoxic stress that promoted acute senescence and in senescent-replicative cultures. Such senotherapeutic activity promoted the recovery of cell proliferation and of MSCs’ stemness properties, and piceatannol clearly exhibited its in vitro antisenescent activity at micromolar concentrations, which were in an order of magnitude of circulating piceatannol after the oral administration of a safe dosage [9,14,16].

### 3.3. Molecular Execution Pathways Associated with Piceatannol’s Antisenescent Activity

The acute senescence of MSCs is primarily associated with P53–P21 and RB1 pathways, while replicative senescence relies on RB2–P16–P21–P27 pathways. The treatment with piceatannol impaired both acute and replicative pathways by significantly reducing the expression of their key signaling proteins. That result suggests that piceatannol can counteract senescence by targeting its associated pathways. In particular, it can affect P53-dependent and -independent senescence. Nevertheless, how piceatannol induces apoptosis in senescent cultures remains to be determined. 

### 3.4. Piceatannol Reinforces SIRT1 Decrease in Senescent Cells

In cells treated with peroxide hydrogen or in replicative senescent cultures, the piceatannol reduced the percentage of senescent cells and promoted a further downregulation of SIRT1 expression compared with healthy cultures. This result is at odds with published data showing that stilbenes, such as resveratrol, protect from senescence through SIRT1 activation [23,24]. It must be considered that the SIRT1 protein can deacetylate and inactivate P53, thus blocking its causative role in apoptosis [31,32]. In our experimental conditions, the increased apoptosis we detected in senescent cultures treated with piceatannol may be ascribed to a reduction in SIRT1 expression that in turn activated a P53-dependent apoptosis process. In this context, the piceatannol may exert its anti-senescence effect through signaling pathways that do not overlap those of resveratrol. There are findings showing that piceatannol and resveratrol may have both common and specific functions [33]. Moreover, to add further complexity, the roles in senescence and apoptosis of such stilbenes are concentration- cell type- and cell status-dependent [13,29,34]. For example, there are studies showing that resveratrol may promote premature senescence by reducing the SIRT1 level [13]. Others showed that piceatannol may have either pro- or anti-apoptotic effect [29]. Our finding showed that in the 1–10 μM range the piceatannol reduced the percentage of senescent cells in MSC cultures following genotoxic stress. It must be evaluated if this stilbene has a similar activity in other stem cell populations and following different genotoxic stimuli. 

### 3.5. Senolytics or Senomorphics?

Preliminary data on cell viability suggest that 1 μM piceatannol acted as a senomorphic agent for cells treated with hydrogen peroxide, since we did not observe any reduction in cell viability despite a decrease in the number of senescent cells. The results of an apoptosis assay revealed that, at a concentration of 1 μM, piceatannol also induced programmed cell death. That discrepancy may be reconciled considering that, at low doses, piceatannol may promote cell proliferation, which suggests that 1 μM piceatannol can kill senescent cells and at once promote the proliferation of nearby healthy cells. Indeed, in replicative senescent cultures, 1 μM piceatannol did not reduce senescence but promoted cell proliferation. Our data are in good agreement with previous findings evidencing that the low concentration of piceatannol may promote cell proliferation of healthy cells [17]. Another study also showed that that piceatannol was cytoprotective in the range between 1 and 10 μM [18]. 

Our results and literature data indicate that the stimulus of proliferation and the antisenescent effects of piceatannol rely on different signaling pathways, as already reported. For example, it has been shown that stilbenes use the PI3K pathway in protection against reactive oxygen species and in order to sustain cell proliferation, while they may promote apoptosis by triggering endoplasmic reticulum stress, Ca^++^ uptake in mitochondria, and the blockage of F-ATPase [14].

### 3.6. Final Remarks

Piceatannol is a promising new senotherapeutic agent that could be useful for treating aging and/or promoting healthy aging. However, future investigations have to evaluate whether piceatannol has the capacity to counteract senescence in other types of cells, especially those belonging to stem cell compartments. Still, other studies need to address the molecular mechanisms that promote apoptosis of senescent cultures in greater depth. Those investigations are a preliminary step before preclinical studies on animal models of aging and its related pathologies.

## 4. Materials and Methods

### 4.1. MSC Cultures

MSCs derived from bone marrow were obtained from the American Type Culture Collection (ATCC PCS-500-012) and grown in alpha-MEM containing 10% FBS, 4 mM L-glutamine, 100 U/mL penicillin–streptomycin, and 5 ng/mL bFGF. We seeded 1.0–2.5 × 10^5^ cells/cm^2^ in alpha-MEM containing 10% FBS and bFGF, and cells were cultivated to 80% confluency. Cells were then further propagated for the assays reported below. After a first group of experiments was performed on healthy MSCs after 7 d of in vitro cultivation (7 DIV), other studies were conducted on the replicative senescent culture at 30 DIV. All cell culture reagents were obtained from Euroclone Life Sciences (Pero, Italy).

### 4.2. Acute and Chronic Senescent MSCs

To induce acute senescence, we treated cells with hydrogen peroxide. Chronic senescent MSCs were obtained by extensive in vitro cultivation for 30 DIV (i.e., replicative senescence) as described elsewhere [21]. During treatment, cells were incubated with 300 μM H_2_O_2_ for 30 min in complete medium, after which the medium was discarded, and the cells were incubated for 48 h in fresh medium before further analysis.

### 4.3. In Situ Senescence-Associated Beta-Gal Assay

The percentage of senescent cells was calculated according to the number of blue, beta–gal-positive cells out of at least 500 cells in different microscope fields, as reported elsewhere [21].

### 4.4. Cell Proliferation and Cytotoxicity Assay

Cell proliferation was determined with Cell Counting Kit-8 (CCK-8) colorimetric assays used to determine cell viability in cell proliferation and cytotoxicity assays (Dojindo Molecular Technologies, Kumamoto, Japan). We seeded 5000 cells in 96-well plates, and CCK-8 assays were performed. Viability was detected by a microplate reader at 450 nm 24 h, 48 h, and 72 h after incubation.

### 4.5. Cell Cycle Analysis

For each analysis, 5 × 10^4^ cells were collected by trypsin treatment and, after being washed with PBS, were fixed in 70% ethanol overnight at −20 °C. The samples were next washed with PBS 1× and finally dissolved in a hypotonic buffer containing propidium iodide (Sigma-Aldrich, Saint Louis, MO, USA). The samples were acquired from a Guava EasyCyte flow cytometer (Merck Millipore, Danvers, MA, USA) and analyzed following a standard procedure using EasyCyte software. The gating strategy we adopted is shown in Supplementary File S2.

### 4.6. Apoptosis Detection

Apoptosis was detected using a fluorescein-conjugated Annexin V kit (Dojindo Molecular Technologies) on a Guava EasyCyte flow cytometer (Merck Millipore) following the manufacturer’s instructions. The kit has two different dyes (Annexin V and 7AAD) to identify apoptotic and non-apoptotic cells. Annexin V binds to phosphatidylserine on apoptotic cells, while 7AAD permeates and stains DNA of late-stage apoptotic and dead cells. The staining procedure allows the identification of 3 cell populations: non-apoptotic cells (Annexin V- and 7AAD-); early apoptotic cells (annexin V+ and 7AAD-); late-apoptotic or dead cells (Annexin V+ and 7AAD+). In our experimental conditions, early and late apoptotic cells were grouped together.

### 4.7. Colony Forming Unit (CFU) Assay 

An aliquot of cells at 7 DIV and 30 DIV was used for the CFU assay. In brief, 1000 cells were plated on 60 mm plates and incubated for 15 d without changing the medium. Once the plates were collected, fixed, and stained with 0.5% crystal violet, the stained colonies were identified under a light microscope and counted.

### 4.8. Western Blot (WB) Analysis 

Cells were lysed in a buffer containing 0.1% Triton (Bio-Rad, Irvine, CA, USA) for 30 min in ice. Next, 20 μg of each lysate was electrophoresed in a polyacrylamide gel and electroblotted onto a nitrocellulose membrane. We used the following primary antibodies: RB1 (AV33212) and GAPDH (G8795) from Sigma-Aldrich; RB2/P130 (R27020) from BD Biosciences (San Jose, CA, USA); p27^KIP1^ (3686) and SIRT1 (9475) from Cell Signaling; p107 (sc-318), p53 (sc-126), and p21^CIP1^ (sc-397) from Santa Cruz Biotechnology (Dallas, TX, USA); and p16^INK4A^ (ab54210) from Abcam (Cambridge, UK). Immunoreactive signals were detected with a horseradish–peroxidase-conjugated secondary antibody (ImmunoReagents, Raleigh, NC, USA) and reacted with ECL plus reagent (Merck Millipore). All of the antibodies were used according to the manufacturer’s instructions. The mean value was quantified densitometrically using Quantity One 1-D analysis software (Bio-Rad).

### 4.9. Statistical Analysis

Statistical significance was performed using one-way ANOVA and *post hoc* tests (TUKEY) in JASP, open-source statistics software supported by the University of Amsterdam (https://jasp-stats.org, accessed on 8 October 2021). All data from statistical analysis appear in Supplementary file S1.

## Figures and Tables

**Table 1:** Cytotoxicity assay.

Piceatannol Treatment	Percentage of Live Cells
CT	100%
0.001 μM PCT	94 ± 4%
0.01 μM PCT	96 ± 2%
0.1 μM PCT	93 ± 3%
1 μM PCT	95 ± 3%
10 μM PCT	92 ± 3% *
100 μM PCT	38 ± 3% ***

MSCs were incubated with increasing concentrations of piceatannol, and cytotoxicity was evaluated after 48 h with the CCK-8 assay (Dojindo Molecular Technologies, Kumamoto, Japan). Data are expressed in arbitrary units and shown with standard deviation (SD), n = 3. The *** *p* < 0.001 and * *p* < 0.05 indicates statistical significance between the control and treated samples.

## Data Availability

Further information regarding data presented in the article may be provided upon request.

## Supplementary Materials

The following are available online at https://www.mdpi.com/article/10.3390/ijms222111619/s1.
