# Supplementary material for: Biomolecular Evaluation of Piceatannol’s Effects in Counteracting the Senescence of Mesenchymal Stromal Cells: A New Candidate for Senotherapeutics?
Source: Int J Mol Sci. 2021 Oct 27;22(21):11619. doi: 10.3390/ijms222111619 (PMC8583715; doi:10.3390/ijms222111619)
Supplement: Supplementary file 1 [file ijms-22-11619-s001.zip › cartella senza nome/Supp File 1.pdf]

Supp File 1

Data Analysis

Results for Table 1

ANOVA

ANOVA - Live Cell

Cases	Sum of Squares	df	Mean Square	F	p
V1	9343.62	6	1557.270	240.5	< .001
Residual	90.67	14	6.476		

Note. Type III Sum of Squares

Post Hoc Tests

Post Hoc Comparisons - V1

		Mean Difference	SE	t	p_{tukey}	Symbol
CT	0.001μM	4.667	2.078	2.246	0.332	
	0.01μM	-4.333	2.078	-2.085	0.410	
	0.1μM	-3.667	2.078	-1.765	0.589	
	1μM	-6.667	2.078	-3.208	0.072	
	10μM	-8.333	2.078	-4.011	0.017	*
	100μM	-64.000	2.078	-30.801	< .001	***

Results Figure 1A

ANOVA

ANOVA - beta-Gal

Cases	Sum of Squares	df	Mean Square	F	p
V1	361.17	5	72.233	37.15	< .001
Residual	23.33	12	1.944		

Note. Type III Sum of Squares

Post Hoc Tests

Post Hoc Comparisons - V1

		Mean Difference	SE	t	p_{tukey}	Symbol
CT	0.001μM	-3.333	1.139	-2.928	0.102	
	0.01μM	-7.333	1.139	-6.441	< .001	***
	0.1μM	-11.333	1.139	-9.954	< .001	***
	1μM	-11.333	1.139	-9.954	< .001	***
	10μM	-11.333	1.139	-9.954	< .001	***

Results Figure 1B

ANOVA

ANOVA - Live Cell

Cases	Sum of Squares	df	Mean Square	F	p
V1	1562.7	5	312.53	17.26	< .001
Residual	217.3	12	18.11		

Note. Type III Sum of Squares

Post Hoc Tests

Post Hoc Comparisons - V1

		Mean Difference	SE	t	p_{tukey}	Symbol
CT	0.001μM	0.667	3.475	0.192	1.000	
	0.01μM	0.333	3.475	0.096	1.000	
	0.1μM	-0.333	3.475	-0.096	1.000	
	1μM	-12.000	3.528	-3.402	0.047	*
	10μM	-25.333	3.475	-7.291	< .001	***

Results Figure 2A

ANOVA

ANOVA - beta-Gal

Cases	Sum of Squares	df	Mean Square	F	p
V1	6221.9	11	565.626	106.1	< .001
Residual	128.0	24	5.333		

Note. Type III Sum of Squares

Post Hoc Tests

Post Hoc Comparisons - V1

		Mean Difference	SE	t	p_{tukey}	Symbol
CT	H	-11.000	1.886	-5.834	< .001	###
	R	-33.667	1.886	-17.854	< .001	###
CT	ABT	-2.667	1.886	-1.414	0.949	
	PCT1μM	2.000	1.886	1.061	0.994	
	PCT10μM	2.667	1.886	1.414	0.949	
H	H+ABT	8.000	1.886	4.243	0.012	**
	PCT1μM	13.000	1.886	6.894	< .001	***
	PCT10μM	13.667	1.886	7.248	< .001	***
R	R+ABT	12.667	1.886	6.718	< .001	***
	R+PCT1μM	3.000	1.886	1.591	0.896	
	R+PCT10μM	12.667	1.886	6.718	< .001	***

Results Figure 2B

ANOVA

ANOVA - Annexin

Cases	Sum of Squares	df	Mean Square	F	p
V1	3966.1	11	360.553	66.22	< .001
Residual	130.7	24	5.444		

Note. Type III Sum of Squares

Post Hoc Tests

Post Hoc Comparisons - V1

		Mean Difference	SE	t	p_{tukey}	Symbol
CT	H	-8.667	1.905	-4.549	0.006	##
	R	5.333	1.905	2.799	0.241	
CT	ABT	0.667	1.905	0.350	1.000	
	PCT1μM	1.776e -15	1.905	9.324e -16	1.000	
	PCT10μM	2.665e -15	1.905	1.399e -15	1.000	
H	H+ABT	-15.333	1.905	-8.048	< .001	***
	H+PCT1μM	-4.667	1.905	-2.449	0.414	
	H+PCT10μM	-17.000	1.905	-8.923	< .001	***
R	R+ABT	-3.333	1.905	-1.750	0.828	
	R+PCT1μM	1.000	1.905	0.525	1.000	
	R+PCT10μM	-1.667	1.905	-0.875	0.999	

Results Figure 3A

ANOVA

ANOVA - 24h CCK-8

Cases	Sum of Squares	df	Mean Square	F	p
V1	0.003	11	3.172e -4	1.730	0.127
Residual	0.004	24	1.833e -4		

Note. Type III Sum of Squares

Post Hoc Tests 24h

Post Hoc Comparisons - V1

		Mean Difference	SE	t	p_{tukey}	Symbol
CT	H	0.023	0.011	2.111	0.620	
	R	0.020	0.011	1.809	0.798	
CT	ABT	0.003	0.011	0.302	1.000	
	PCT1μM	-0.010	0.011	-0.905	0.998	
	PCT10μM	1.110e -16	0.011	1.004e -14	1.000	
H	H+ABT	-0.027	0.011	-2.412	0.434	
	H+PCT1μM	-0.027	0.011	-2.412	0.435	
	H+PCT10μM	-0.010	0.011	-0.905	0.998	
R	R+ABT	-0.013	0.011	-1.206	0.983	
	R+PCT1μM	-0.020	0.011	-1.809	0.798	
	R+PCT10μM	-0.010	0.011	-0.905	0.998	

ANOVA

ANOVA - 48 CCK-8

Cases	Sum of Squares	df	Mean Square	F	p
V1	0.911	11	0.083	26.91	< .001
Residual	0.074	24	0.003		

Note. Type III Sum of Squares

Post Hoc Tests 48h

Post Hoc Comparisons - V1

			Mean Difference	SE	t	p_{tukey}	Symbol
48h	CT	H	0.183	0.045	4.047	0.018	##
		R	0.443	0.045	9.787	< .001	###
	CT	ABT	0.100	0.045	2.208	0.560	
		PCT1μM	-0.050	0.045	-1.104	0.991	

Post Hoc Comparisons - V1

		Mean Difference	SE	t	p_{tukey}	Symbol
	PCT10μM	-0.030	0.045	-0.662	1.000	
H	H+ABT	-0.093	0.045	-2.060	0.652	
	H+PCT1μM	-0.047	0.045	-1.030	0.995	
	H+PCT10μM	-0.087	0.045	-1.913	0.741	
R	R+ABT	-0.117	0.045	-2.576	0.345	
	R+PCT1μM	-0.150	0.045	-3.311	0.093	*
	R+PCT10μM	-0.230	0.045	-5.078	0.002	**

ANOVA

ANOVA - 72 CCK-8

Cases	Sum of Squares	df	Mean Square	F	p
V1	51.654	11	4.696	205.6	< .001
Residual	0.548	24	0.023		

Note. Type III Sum of Squares

Post Hoc Tests

Post Hoc Comparisons - V1

		Mean Difference	SE	t	p_{tukey}	Symbol
CT	H	2.100	0.123	17.020	< .001	###
	R	3.083	0.123	24.989	< .001	###
CT	ABT	0.300	0.123	2.431	0.424	
	PCT1μM	0.033	0.123	0.270	1.000	
	PCT10μM	0.067	0.123	0.540	1.000	
H	H+ABT	-1.200	0.123	-9.726	< .001	***
	H+PCT1μM	-0.233	0.123	-1.891	0.753	
	H+PCT10μM	-0.400	0.123	-3.242	0.107	
R	R+ABT	-0.383	0.123	-3.107	0.138	
	R+PCT1μM	-0.703	0.123	-5.700	< .001	***
	R+PCT10μM	-1.017	0.123	-8.240	< .001	***

Results figure 3B

ANOVA

ANOVA - Ki67

Cases	Sum of Squares	df	Mean Square	F	p
V1	1862.8	11	169.34	11.04	< .001
Residual	368.0	24	15.33		

Note. Type III Sum of Squares

Post Hoc Tests

Post Hoc Comparisons - V1

		Mean Difference	SE	t	p_{tukey}	Symbol
CT	H	6.000	3.197	1.877	0.762	
	R	21.000	3.197	6.568	< .001	###
CT	ABT	-0.667	3.197	-0.209	1.000	
	PCT1µM	-3.667	3.197	-1.147	0.988	
	PCT10µM	2.333	3.197	0.730	1.000	
H	H+ABT	-2.333	3.197	-0.730	1.000	
	H+PCT1µM	-5.333	3.197	-1.668	0.866	
	H+PCT10µM	-7.667	3.197	-2.398	0.443	
R	R+ABT	-17.667	3.197	-5.526	0 .011	**
	R+PCT1µM	-8.667	3.197	-2.711	0.009	**
	R+PCT10µM	-28.667	3.197	-8.966	< .001	***

Results figure 3C

ANOVA

ANOVA - G1 Cell Cycle

Cases	Sum of Squares	df	Mean Square	F	p
V1	3457.6	11	314.331	32.52	< .001
Residual	232.0	24	9.667		

Note. Type III Sum of Squares

Post Hoc Tests

Post Hoc Comparisons - V1

		Mean Difference	SE	t	p_{tukey}	Symbol
CT	H	-7.333	2.539	-2.889	0.207	
	R	-21.000	2.539	-8.272	< .001	###
CT	ABT	2.667	2.539	1.050	0.994	
	PCT1μM	-0.333	2.539	-0.131	1.000	
	PCT10μM	-1.333	2.539	-0.525	1.000	
H	H+ABT	20.667	2.539	8.141	< .001	***
	H+PCT1μM	23.333	2.539	9.191	< .001	***
	H+PCT10μM	14.333	2.539	5.646	< .001	***
R	R+ABT	20.333	2.539	8.010	< .001	***
	R+PCT1μM	10.667	2.539	4.202	0.013	*
	R+PCT10μM	28.667	2.539	11.292	< .001	***

ANOVA

ANOVA - S Cell Cycle

Cases	Sum of Squares	df	Mean Square	F	p
V1	1802.3	11	163.85	15.94	< .001
Residual	246.7	24	10.28		

Note. Type III Sum of Squares

Post Hoc Tests

Post Hoc Comparisons - V1

		Mean Difference	SE	t	p_{tukey}	Symbol
CT	H	9.333	2.618	3.566	0.055	
	R	16.667	2.618	6.367	< .001	###
CT	ABT	-1.667	2.618	-0.637	1.000	
	PCT1μM	-9.333	2.618	-3.566	0.055	
	PCT10μM	-3.667	2.618	-1.401	0.952	
H	H+ABT	-13.333	2.618	-5.094	0.002	*
	H+PCT1μM	-13.333	2.618	-5.094	0.001	**
	H+PCT10μM	-13.667	2.618	-5.221	0.001	**
R	R+ABT	-9.000	2.618	-3.438	0.071	
	R+PCT1μM	-9.667	2.618	-3.693	0.042	
	R+PCT10μM	-15.667	2.618	-5.985	< .001	***

ANOVA

ANOVA - G2/M Cell Cycle

Cases	Sum of Squares	df	Mean Square	F	p
V1	1813.3	11	164.85	15.41	< .001
Residual	256.7	24	10.69		

Note. Type III Sum of Squares

Post Hoc Tests

Post Hoc Comparisons - V1

		Mean Difference	SE	t	p_{tukey}	Symbol
CT	H	-2.667	2.670	-0.999	0.996	
	R	4.333	2.670	1.623	0.884	
CT	ABT	5.000	2.670	1.873	0.764	
	PCT1μM	-4.333	2.670	-1.623	0.884	
	PCT10μM	-5.333	2.670	-1.997	0.691	
H	H+ABT	-7.000	2.670	-2.622	0.321	
	H+PCT1μM	-9.000	2.670	-3.371	0.082	
	H+PCT10μM	-6.667	2.670	-2.497	0.386	
R	R+ABT	-22.667	2.670	-8.489	< .001	***
	R+PCT1μM	-16.667	2.670	-6.242	< .001	***
	R+PCT10μM	-26.000	2.670	-9.737	< .001	***

Results figure 4

ANOVA

ANOVA - CFU

Cases	Sum of Squares	df	Mean Square	F	p
V1	6.258e +6	11	568871	55.86	< .001
Residual	244431	24	10185		

Note. Type III Sum of Squares

Post Hoc Tests

Post Hoc Comparisons - V1

		Mean Difference	SE	t	p_{tukey}	Symbol
CT	H	1076.667	82.40	13.066	< .001	###
	R	1067.000	82.40	12.949	< .001	###
CT	ABT	-98.333	82.40	-1.193	0.985	
	PCT1μM	-66.000	82.40	-0.801	0.999	
	PCT10μM	47.000	82.40	0.570	1.000	
H	H+ABT	-219.000	82.40	-2.658	0.304	
	H+PCT1μM	-132.667	82.40	-1.610	0.889	
	H+PCT10μM	-145.000	82.40	-1.760	0.823	
R	R+ABT	-501.000	82.40	-6.080	< .001	***
	R+PCT1μM	-568.000	82.40	-6.893	< .001	***
	R+PCT10μM	-584.667	82.40	-7.095	< .001	***

Results Figure 5

ANOVA

ANOVA – RB1

Cases	Sum of Squares	df	Mean Square	F	p
V1	33.905	11	3.082	24.00	< .001
Residual	3.083	24	0.128		

Note. Type III Sum of Squares

Post Hoc Tests

Post Hoc Comparisons - V1

		Mean Difference	SE	t	p_{tukey}	Symbol
CT	H	-1.513	0.293	-5.172	0.001	##
	R	1.610	0.293	5.502	< .001	###
CT	ABT	-0.540	0.293	-1.845	0.779	
	PCT1μM	0.817	0.293	2.791	0.245	
	PCT10μM	1.600	0.293	5.468	< .001	***
H	H+ABT	2.337	0.293	7.985	< .001	***
	H+PCT1μM	2.933	0.293	10.024	< .001	***
	H+PCT10μM	2.590	0.293	8.851	< .001	***
R	R+ABT	0.643	0.293	2.199	0.564	
	R+PCT1μM	-0.207	0.293	-0.706	1.000	
	R+PCT10μM	0.420	0.293	1.435	0.944	

ANOVA

ANOVA - RB2

Cases	Sum of Squares	df	Mean Square	F	p
V1	1.003	11	0.091	39.68	< .001
Residual	0.055	24	0.002		

Note. Type III Sum of Squares

Post Hoc Tests

Post Hoc Comparisons - V1

		Mean Difference	SE	t	p_{tukey}	Symbol
CT	H	-0.057	0.039	-1.448	0.941	
	R	-0.220	0.039	-5.622	< .001	###
CT	ABT	0.433	0.039	11.073	< .001	***
	PCT1μM	-0.197	0.039	-5.025	0.002	**
	PCT10μM	-0.283	0.039	-7.240	< .001	***
H	H+ABT	0.057	0.039	1.448	0.941	
	H+PCT1μM	0.183	0.039	4.685	0.004	**
	H+PCT10μM	0.170	0.039	4.344	0.009	**
R	R+ABT	0.073	0.039	1.874	0.763	
	R+PCT1μM	0.217	0.039	5.537	< .001	***
	R+PCT10μM	0.317	0.039	8.092	< .001	***

ANOVA

ANOVA - P107

Cases	Sum of Squares	df	Mean Square	F	p
V1	1.694	11	0.154	47.82	< .001
Residual	0.077	24	0.003		

Note. Type III Sum of Squares

Post Hoc Tests

Post Hoc Comparisons - V1

		Mean Difference	SE	t	p_{tukey}	Symbol
CT	H	-0.110	0.046	-2.374	0.457	
	R	0.370	0.046	7.987	< .001	###
CT	ABT	0.060	0.046	1.295	0.972	
	PCT1μM	-0.300	0.046	-6.476	< .001	***
	PCT10μM	-0.013	0.046	-0.288	1.000	
H	H+ABT	0.350	0.046	7.555	< .001	***
	H+PCT1μM	0.530	0.046	11.440	< .001	***
	H+PCT10μM	0.493	0.046	10.649	< .001	***
R	R+ABT	-0.083	0.046	-1.799	0.804	
	R+PCT1μM	-0.293	0.046	-6.332	< .001	***
	R+PCT10μM	-0.180	0.046	-3.885	0.027	*

ANOVA

ANOVA - P53

Cases	Sum of Squares	df	Mean Square	F	p
V1	125.971	11	11.452	59.60	< .001
Residual	4.611	24	0.192		

Note. Type III Sum of Squares

Post Hoc Tests

Post Hoc Comparisons - V1

		Mean Difference	SE	t	p_{tukey}	Symbol
CT	H	-4.017	0.358	-11.223	< .001	###
	R	0.250	0.358	0.699	1.000	
ABT	CT	1.270	0.358	3.549	0.056	
	PCT1μM	0.060	0.358	0.168	1.000	
	PCT10μM	0.690	0.358	1.928	0.733	
H	H+ABT	4.200	0.358	11.735	< .001	***
	H+PCT1μM	2.993	0.358	8.364	< .001	***
	H+PCT10μM	1.970	0.358	5.504	< .001	***
R	R+ABT	0.997	0.358	2.785	0.248	
	R+PCT1μM	3.787	0.358	10.580	< .001	***
	R+PCT10μM	0.573	0.358	1.602	0.893	

ANOVA

ANOVA - P27

Cases	Sum of Squares	df	Mean Square	F	p
V1	24.723	11	2.248	19.79	< .001
Residual	2.726	24	0.114		

Note. Type III Sum of Squares

Post Hoc Tests

Post Hoc Comparisons - V1

		Mean Difference	SE	t	p_{tukey}	Symbol
CT	H	-0.013	0.275	-0.048	1.000	
	R	-0.360	0.275	-1.308	0.970	
CT	ABT	0.530	0.275	1.926	0.733	
	PCT1μM	-0.190	0.275	-0.690	1.000	
	PCT10μM	-0.930	0.275	-3.380	0.081	
H	H+ABT	0.830	0.275	3.016	0.165	
	H+PCT1μM	0.143	0.275	0.521	1.000	
	H+PCT10μM	-0.220	0.275	-0.800	0.999	
R	R+ABT	2.027	0.275	7.365	< .001	***
	R+PCT1μM	0.540	0.275	1.962	0.712	
	R+PCT10μM	2.337	0.275	8.492	< .001	***

ANOVA

ANOVA - P21

Cases	Sum of Squares	df	Mean Square	F	p
V1	4.902	11	0.446	16.34	< .001
Residual	0.655	24	0.027		

Note. Type III Sum of Squares

Post Hoc Tests

Post Hoc Comparisons - V1

		Mean Difference	SE	t	p_{tukey}	Symbol
CT	H	-0.740	0.135	-5.488	< .001	###
	R	-1.087	0.135	-8.059	< .001	###
ABT	CT	-0.163	0.135	-1.211	0.983	
	PCT1μM	0.077	0.135	0.569	1.000	
	PCT10μM	0.137	0.135	1.014	0.996	
H	H+ABT	0.257	0.135	1.904	0.746	
	H+PCT1μM	0.417	0.135	3.090	0.144	
	H+PCT10μM	0.233	0.135	1.730	0.837	
R	R+ABT	0.600	0.135	4.450	0.007	**
	R+PCT1μM	0.497	0.135	3.683	0.043	*
	R+PCT10μM	0.610	0.135	4.524	0.006	**

ANOVA

ANOVA - P16

Cases	Sum of Squares	df	Mean Square	F	p
V1	7.998	11	0.727	20.83	< .001
Residual	0.838	24	0.035		

Note. Type III Sum of Squares

Post Hoc Tests

Post Hoc Comparisons - V1

		Mean Difference	SE	t	p_{tukey}	Symbol
CT	H	-0.533	0.153	-3.496	0.063	
	R	-0.670	0.153	-4.392	0.008	##
CT	ABT	0.023	0.153	0.153	1.000	
	PCT1μM	0.257	0.153	1.683	0.859	
	PCT10μM	0.667	0.153	4.371	0.009	**
H	H+ABT	0.013	0.153	0.087	1.000	
	H+PCT1μM	-0.127	0.153	-0.830	0.999	
	H+PCT10μM	-0.037	0.153	-0.240	1.000	
R	R+ABT	0.767	0.153	5.026	0.002	**
	R+PCT1μM	0.947	0.153	6.206	< .001	***
	R+PCT10μM	1.300	0.153	8.523	< .001	***

ANOVA

ANOVA - SIRT1

Cases	Sum of Squares	df	Mean Square	F	p
V1	41.807	11	3.801	86.94	< .001
Residual	1.049	24	0.044		

Note. Type III Sum of Squares

Post Hoc Tests

Post Hoc Comparisons - V1

		Mean Difference	SE	t	p_{tukey}	Symbol
CT	H	1.467	0.171	8.591	< .001	###
	R	1.300	0.171	7.615	< .001	###
CT	ABT	0.023	0.171	0.137	1.000	
	PCT1μM	0.490	0.171	2.870	0.213	
	PCT10μM	0.443	0.171	2.597	0.334	
H	H+ABT	1.067	0.171	6.248	< .001	***
	H+PCT1μM	1.367	0.171	8.005	< .001	***
	H+PCT10μM	1.653	0.171	9.685	< .001	***
R	R+ABT	0.367	0.171	2.148	0.597	
	R+PCT1μM	0.250	0.171	1.464	0.936	
	R+PCT10μM	1.500	0.171	8.786	< .001	***
