# Supplementary figures and images for: Biomolecular Evaluation of Piceatannol’s Effects in Counteracting the Senescence of Mesenchymal Stromal Cells: A New Candidate for Senotherapeutics?
Source: Int J Mol Sci. 2021 Oct 27;22(21):11619. doi: 10.3390/ijms222111619 (PMC8583715; doi:10.3390/ijms222111619)

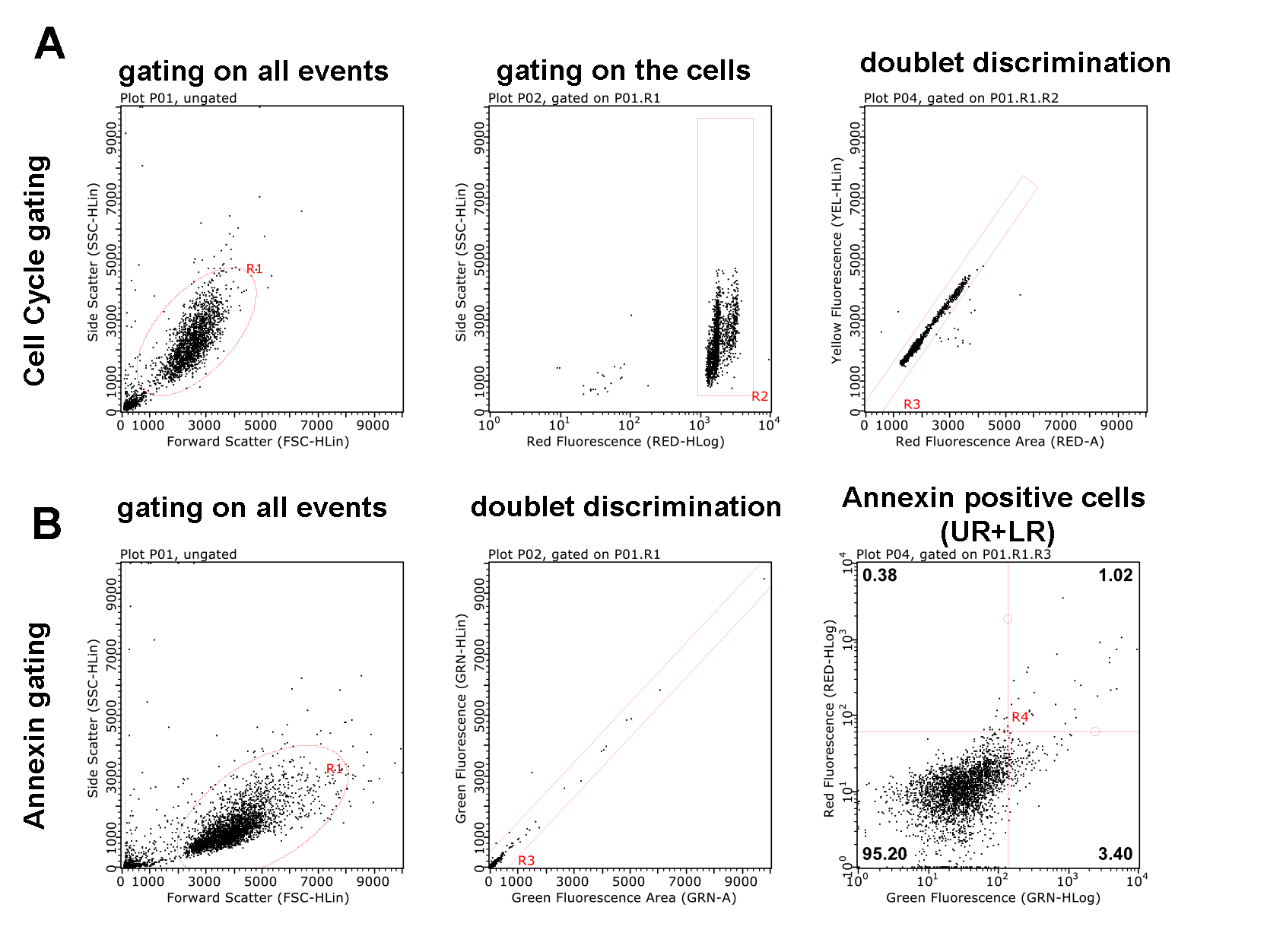

Supplement: Supplementary file 1 [file ijms-22-11619-s001.zip › cartella senza nome/Supp file 2.tif]

# Supp File 3

Raw data WB images



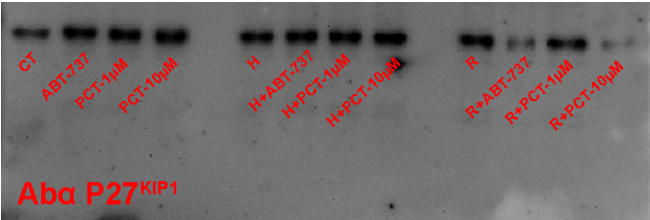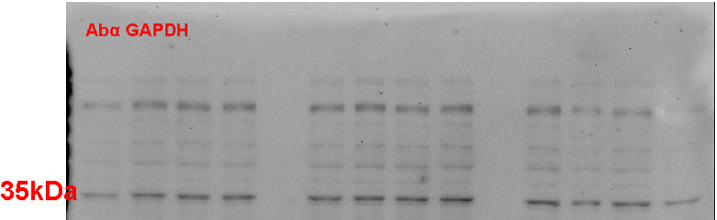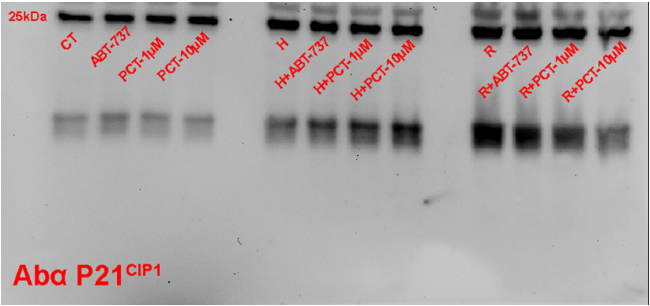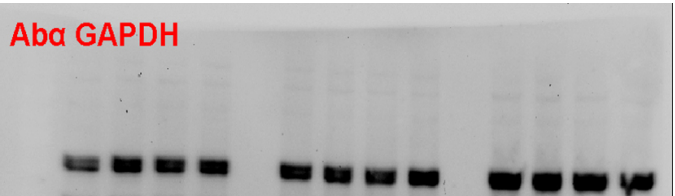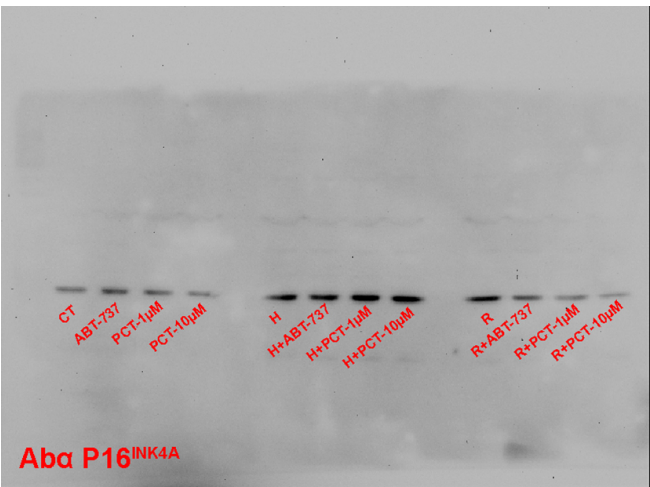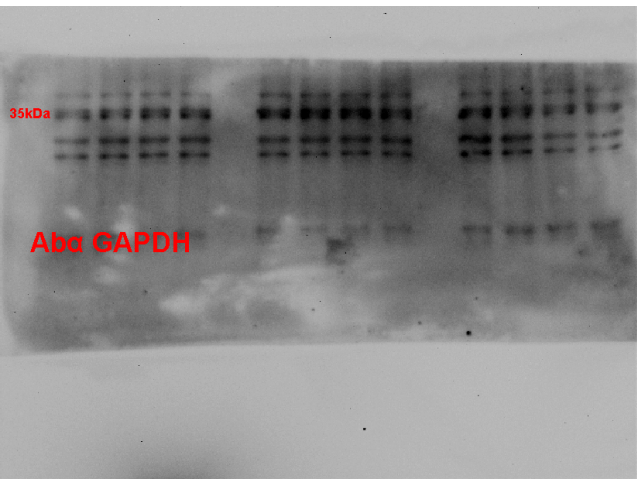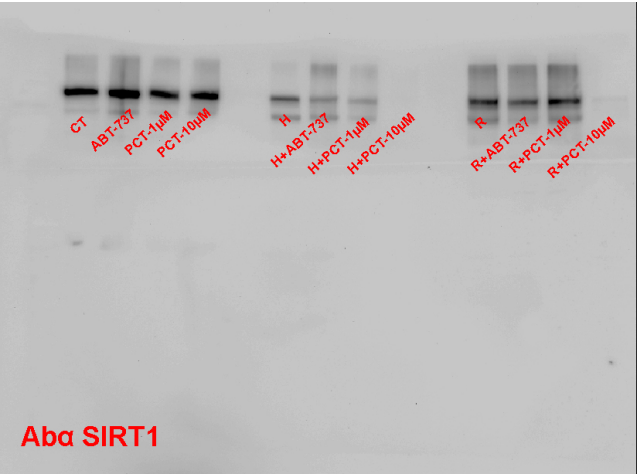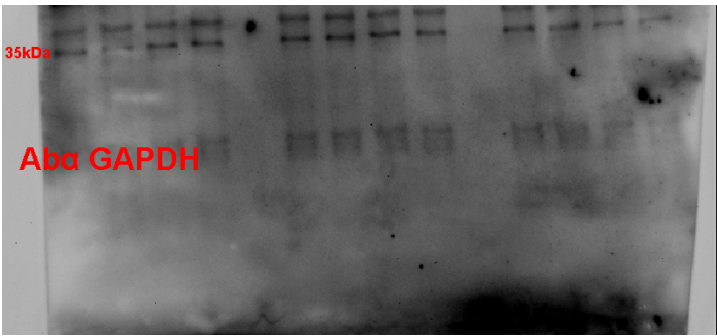

Supplement: Supplementary file 1 [file ijms-22-11619-s001.zip › cartella senza nome/Supp File 3.pdf]
